# Preoperative prediction of microvascular/nerve invasion in locally advanced gastric cancer by differentiation and enhanced CT features

**DOI:** 10.1097/MD.0000000000040816

**Published:** 2024-12-13

**Authors:** Xiu-Chun Ren, Pan Liang

**Affiliations:** a Department of Ultrasonography, The First Affiliated Hospital, Zhengzhou University, Zhengzhou, China; b Department of Radiology, The First Affiliated Hospital, Zhengzhou University, Zhengzhou, China.

**Keywords:** gastric cancer, influencing factors, microvascular/nerve invasion, prediction, tomography, X-ray computed

## Abstract

The purpose of the article is to determine whether differentiation and enhanced CT features can preoperatively predict microvascular/nerve invasion in locally advanced gastric cancer. Retrospective analysis of the CT and pathological data of 325 patients with locally advanced gastric cancer confirmed by pathology in our hospital from July 2011 to August 2023. The patient’s age, gender, tumor location, T stage, N stage, TNM stage, differentiation, Lauren classification, as well as tumor thickness, tumor longest diameter, plain CT value, arterial CT value, venous CT value, arterial phase enhancement rate, and venous phase enhancement rate were assessed. This study included a total of 325 patients with locally advanced gastric cancer and 189 patients (58.15%) with microvascular/nerve invasion. The results of the univariate analysis showed that gender, location, T stage, N stage, TNM stage, differentiation, Lauren classification, tumor thickness, and longest diameter of the tumor were associated with microvascular/nerve invasion (*P* < .05). Multivariate analysis suggested that TNM stage and differentiation were independent risk factors for microvascular/nerve invasion. The receiver operating characteristic analysis showed that the diagnostic efficacy of the combined parameter of TNM stage and differentiation was better than that of the single parameter, in which area under the curve, sensitivity, and specificity were 0.819 (95%CI: 0.770–0.867), 66.7%, and 83.8%, respectively. Differentiation and enhanced CT are helpful in predicting whether microvascular/nerve invasion occurs in locally advanced gastric cancer before operation, especially the combined parameters of TNM stage and differentiation.

## 1. Introduction

Gastric cancer is one of the most common malignant tumors in the digestive system, with onset hidden, rapid progression, and poor treatment effectiveness.^[1]^ Although patients with locally advanced gastric cancer can receive radical treatment through surgical resection at the early stage, the recurrence rate after surgery is as high as 42.5%, and the long-term prognosis of patients is still not ideal.^[2]^ Postoperative recurrence of locally advanced gastric cancer has become the main cause of death in patients, related to tumor size, differentiation, pathological type, and TNM stage.^[3]^ In addition, multiple studies have confirmed that microvascular/nerve invasion is an independent risk factor for recurrence and metastasis of locally advanced gastric cancer,^[2,4]^ and an independent predictor of postoperative disease-free survival and overall survival.^[5,6]^ Consequently, accurately determining the microvascular/nerve invasion status of locally advanced gastric cancer before surgery is the key to formulating personalized treatment strategies. At present, enhanced CT has become a routine examination method for the preoperative evaluation of gastric cancer, but previous studies have mostly focused on subjective morphological analysis of gastric cancer.^[2–4]^ Currently, there are few reports on the predictive value of differentiation and enhanced CT examination for microvascular/nerve invasion in locally advanced gastric cancer both domestically and internationally, and there are conflicting results.^[2,4–6]^ Based on a retrospective analysis of the clinical and pathological data of 325 locally advanced gastric cancer patients, this study explores the application value of differentiation and enhanced CT values in the preoperative prediction of microvascular/nerve invasion in locally advanced gastric cancer.

## 2. Materials and methods

### 2.1. Subjects

The retrospective study was approved by the Institutional Review Board of the first Hospital of Zhengzhou University. The written informed consent was waived.

### 2.2. Patients and study design

The data of patients with locally advanced gastric cancer diagnosed by pathology after radical surgical resection in our hospital from July 2011 to August 2023 were retrospectively collected. Inclusion criteria were as follows: (a) radical surgical resection, (b) pathologically confirmed locally advanced gastric cancer with microvascular/nerve invasion results, (c) abdominal CT enhanced examination was performed within 2 weeks before surgery. Exclusion criteria for patients were as follows: (a) receiving other nonsurgical treatments before surgery, including radiotherapy, chemotherapy, or radiochemotherapy, (b) small lesions or poor image quality were difficult to evaluate, (c) incomplete clinical and pathological data. Microvascular invasion means that tumor cells break through mucosal epithelium and invade blood vessels and lymphatic vessels.^[4,5]^ Nerve invasion refers to the phenomenon that tumor cells invade any layer of the endoneurium, neurilemma, or epineurium, or invade more than 1/3 of the peripheral diameter of the nerve, and infiltrate and metastasize locally along the nerve fibers, that is, tumor perineural invasion or perineural invasion.^[2,5,6]^

**Figure 1.**
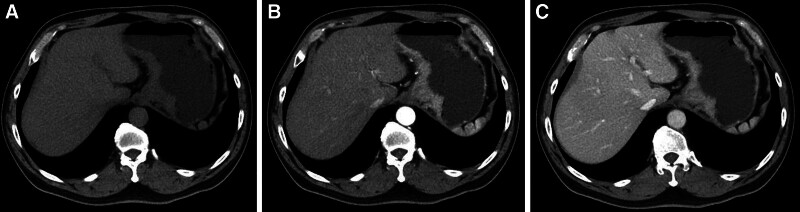
A typical case (male, 58 years old, microvascular invasion (+) and nerve invasion (+)) with local gastric wall thickening with abnormal enhancement on CT (T3 stage, N3 stage, adenocarcinoma, poor differentiated, intestinal type). (A) Plain CT image: local gastric wall thickening in the gastric cardia. (B and C) Enhanced CT image: the thickened gastric wall in the gastric cardia is significantly enhanced.

**Figure 2.**
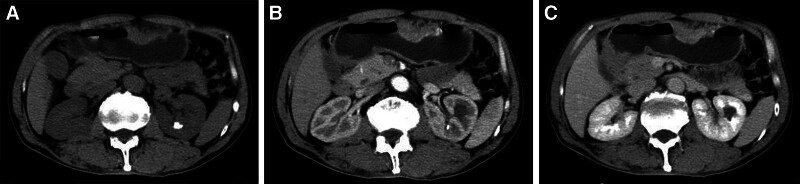
A typical case (male, 64 years old, microvascular invasion (+)) with local gastric wall thickening with abnormal enhancement on CT (T3 stage, N3 stage, adenocarcinoma, nonpoor differentiated, diffuse type). (A) Plain CT image: local gastric wall thickening in the gastric body. (B and C) Enhanced CT image: the thickened gastric wall in the gastric body is significantly enhanced.

**Figure 3.**
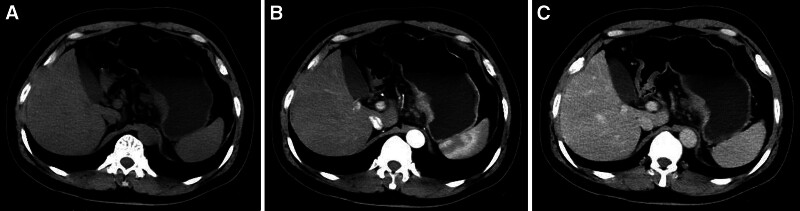
A typical case (male, 52 years old, nerve invasion (+)) with local gastric wall thickening with abnormal enhancement on CT (T3 stage, N0 stage, adenocarcinoma, nonpoor differentiated, mixed type). (A) Plain CT image: local gastric wall thickening in the gastric cardia. (B and C) Enhanced CT image: the thickened gastric wall in the gastric cardia is significantly enhanced.

**Figure 4.**
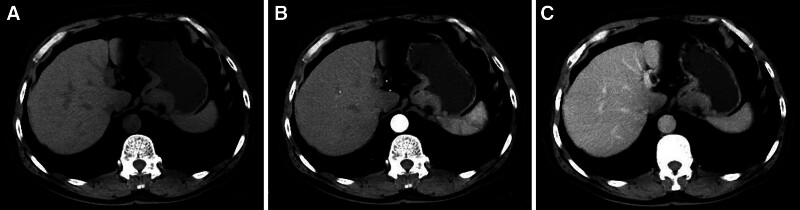
A typical case (male, 62 years old, microvascular/nerve invasion (-)) with local gastric wall thickening with abnormal enhancement on CT (T3 stage, N3 stage, adenocarcinoma, nonpoor differentiated, mixed type). (A) Plain CT image: local gastric wall thickening in the gastric cardia. (B and C) Enhanced CT image: the thickened gastric wall in the gastric cardia is significantly enhanced.

Based on the above inclusion criteria, a total of 325 patients with locally advanced gastric cancer were collected, including 238 males (73.23%) and 87 females (26.77%), aged 25 to 86 years (60.59 ± 10.847). Based on the findings of intraoperative exploration and postoperative pathological examination, 189 cases (58.15%) in the microvascular/nerve invasion group and 136 cases (41.85%) in the non-microvessel/nerve invasion group were found (Figs. 1–4).

### 2.3. CT study protocols

Take 600 to 1000 mL of water orally to distend the stomach, followed by 40 mg of butyl scopolamine to reduce bowel peristalsis and promote hypotonia. The Discovery CT 750 HD scanner (GE Healthcare, Wisconsin) was used, the tube voltage was 100 kVp, the tube current was automatically selected by mA, the pitch was 1.375, the detector width was 0.625 mm × 64, and the reconstructed slice thickness was 5 mm. A contrast agent (370 mgI/mL of Univision) was injected through the cubital vein with a high-pressure syringe, the flow rate was 3.5 mL/s, and the dose was 1.5 mL/kg body weight. Arterial phase and venous phase scans were performed 25 to 30 seconds and 60 seconds after the injection of the contrast agent, respectively.

### 2.4. Clinical characteristics and radiologic assessment

The data on clinical characteristics collected through electronic health records included age, gender, tumor location, T stage, N stage, TNM stage, differentiation, and Lauren classification. CT examination data: tumor thickness, longest diameter, plain CT value, arterial phase CT value, venous phase CT value, arterial phase enhancement rate, venous phase enhancement rate. The tumor location was divided into gastric cardia and fundus, gastric body, gastric antrum, and pylorus according to its anatomical location. CT values of the lesion were calculated in Hounsfield units. Tumor thickness was calculated as the largest diameter of the tumor perpendicular to the base of the tumor. The longest diameter was calculated according to the maximum length of the tumor parallel to the base of the tumor. The arterial phase enhancement rate refers to (the arterial phase CT value-plain CT value)/plain CT value and the venous phase enhancement rate refers to (the venous phase CT value-plain CT value)/plain CT value. A circular region of interest was placed in the tumor, as large as possible to reduce noise (0.50 pixels), away from any peripheral fat and necrotic area.

### 2.5. Statistical analyses

Statistical analyses were performed by SPSS software version 21.0. The independent samples *t* test was used for quantitative data when it conformed to the normal distribution. The Mann–Whitney *U* test was used when it did not conform to the normal distribution, and the chi-square test was used for qualitative data. Statistically significant variables were analyzed using a binary logistic regression model. Draw receiver operating characteristic (ROC) curves for parameters with differences in logistic regression model analysis, and calculate the area under the curve (AUC) to test the diagnostic efficacy of each parameter. The DeLong test was used to compare parameter performance. *P* < .05 indicated a statistically significant difference.

## 3. Results

### 3.1. Univariate analysis results of microvascular/nerve invasion in locally advanced gastric cancer

Table 1 of the univariate analysis showed that gender, location, T stage, N stage, TNM stage, differentiation, Lauren classification, tumor thickness, and longest diameter of the tumor were correlated with microvascular/nerve invasion, all *P* < .05 (Figs. 1–4).

**Table 1:** Univariate analysis results of microvascular/nerve invasion in locally advanced gastric cancer patients.

Variables	Non-microvascular/nerve invasion (136 cases)	Microvascular/nerve invasion (189 cases)	Statistics value	*P* value
Gender [(%)]			6.984	.008**
Male	110 (80.88)	128 (67.72)		
Female	26 (19.12)	61 (32.28)		
Age [(%)]			0.414	.520
≤60	57 (41.91)	86 (45.50)		
>60	79 (58.09)	103 (54.50)		
Location [(%)]			6.682	.035*
Gastric cardia and fundus	79 (58.09)	84 (44.44)		
Gastric body	22 (16.18)	33 (17.46)		
Gastric antrum and angle	35 (25.74)	72 (38.10)		
T stage [(%)]			54.336	.000**
T2	47 (34.56)	12 (6.35)		
T3	77 (56.62)	116 (61.38)		
T4	12 (8.82)	61 (32.28)		
N stage [(%)]			68.297	.000**
N0	72 (52.94)	23 (12.17)		
N1	19 (13.97)	40 (21.16)		
N2	20 (14.71)	33 (17.46)		
N3	25 (18.38)	93 (49.21)		
TNM stage [(%)]			80.034	.000**
I	35 (25.74)	3 (1.59)		
II	50 (36.76)	29 (15.34)		
III/IV	51 (37.50)	157 (83.07)		
Differentiation [(%)]			62.809	.000**
Nonpoor differentiated	88 (64.71)	40 (21.16)		
Poor differentiation	48 (35.29)	149 (78.84)		
Lauren classification [(%)]			12.113	.002**
Intestinal type	70 (51.47)	61 (32.28)		
Diffuse type	22 (16.18)	43 (22.75)		
Mixed type	44 (32.35)	85 (44.97)		
Thickness [mm, mean ± SD]	15.09 ± 5.98	16.17 ± 5.48	-1.690	.092
Longest tumor diameter [mm, mean ± SD]	47.03 ± 17.16	54.06 ± 15.93	-3.798	.000**
Plain CT value [Hu, mean ± SD]	38.14 ± 7.85	37.97 ± 7.05	0.208	.835
Arterial phase CT value [Hu, mean ± SD]	73.11 ± 17.93	71.28 ± 18.85	0.881	.379
Venous phase CT value [Hu, mean ± SD]	83.21 ± 20.16	84.83 ± 18.61	-0.746	.456
Arterial phase enhancement rate [mean ± SD]	0.97 ± 0.51	0.92 ± 0.56	0.792	.429
Venous phase enhancement rate [mean ± SD]	1.24 ± 0.57	1.29 ± 0.60	-0.794	.428

Hu = Hounsfeld units.

* *P* < .05.

** *P* < .01.

### 3.2. Multivariate analysis results of microvascular/nerve invasion in locally advanced gastric cancer

The logistic regression model analysis of variables with statistical differences in the univariate analysis showed that TNM stage and differentiation were independent risk factors for microvascular/nerve invasion in locally advanced gastric cancer (Table 2).

**Table 2:** Multivariate analysis results of microvascular/nerve invasion in locally advanced gastric cancer patients.

Variables	OR value	95%CI	*P* value
TNM stage	2.751	1.246–6.072	.012
Differentiation	4.961	2.689–9.155	.000

### 3.3. Diagnostic efficacy of microvascular/nerve invasion in locally advanced gastric cancer

The ROC of microvascular/nerve invasion of locally advanced gastric cancer shows that the diagnostic efficacy of TNM stage and differentiation is good (AUC value is 0.745 and 0.718), and the diagnostic efficacy of combined parameter is good (AUC value is 0.819, 95%CI 0.770–0.867) (Table 3). The DeLong test results showed that the AUC of the combined parameter was superior to the diagnostic efficacy of TNM stage and differentiation (*P* < .05).

**Table 3:** Diagnostic efficacy of microvascular/nerve invasion in locally advanced gastric cancer.

Variables	AUC (95%CI)	Youden index	Optimal threshold	Sensitivity (%)	Specificity (%)
TNM stage	0.745 (0.688–0.802)	0.456	2.000	83.1	62.5
Differentiation	0.718 (0.660–0.776)	0.435	2.000	78.8	64.7
Combined parameter	0.819 (0.770–0.867)	0.505	0.547	66.7	83.8

AUC = area under the curve.

## 4. Discussion

Locally advanced gastric cancer has been transformed into a multidisciplinary cooperative comprehensive treatment mode centered on surgery. Combined with radiotherapy, chemotherapy, molecular targeted therapy, etc, the survival period of patients has been extended to a certain extent. Microvascular/nerve invasion is an important marker related to the malignant biological behavior and prognosis of locally advanced gastric cancer and has guiding significance for the development of personalized treatment strategies and intervention and management. Moreover, it is difficult to provide sufficient information for the prognosis of gastric cancer patients based on postoperative histological classification and the TNM stage.^[7–9]^ Because of this, early detection of microvascular/nerve invasion can help supplement the clinical value of the TNM stage. However, the current diagnostic basis for microvascular/nerve invasion still requires postoperative pathological examination, which lacks guidance for the preoperative management of locally advanced gastric cancer patients.

Gabbert et al^[8]^ showed that the incidence of vascular invasion in lymph node-positive patients is higher than that in lymph node-negative patients, and the 5-year survival rate of patients with vascular invasion is significantly reduced. A study on patients undergoing radical gastrectomy showed that,^[6]^ the incidence of perineural invasion in gastric cancer patients undergoing radical gastrectomy was high, and the proportion of positive perineural invasion increased with disease progression and clinical stage. Neural invasion was related to prognosis after radical gastrectomy, and suggested poor prognosis. It is worth mentioning that previous studies have mostly focused on pathological diagnostic methods for microvascular or neural invasion,^[10]^ as well as the value of single features of microvascular or neural invasion in evaluating the prognosis of gastric cancer patients. Given that 58.15% of locally advanced gastric cancer patients in this study have microvascular invasion accompanied by neural invasion, the author emphasizes that “microvascular invasion” is accompanied by “neural invasion.”

Research^[4,8,11,12]^ has shown that microvascular/nerve invasion in gastric cancer patients is associated with depth of gastric wall invasion, TNM stage, tumor diameter, and lymph node metastasis. In this study, the incidence of microvascular/nerve invasion in T3 and T4 locally advanced gastric cancer patients was 93.65%, significantly higher than that in T2 gastric cancer patients; the average tumor diameter of patients in the microvascular/nerve invasion group was 54.06 mm, higher than the 47.03 mm in the non-microvascular/nerve invasion group. The results of multivariate analysis showed that TNM stage and differentiation are independent risk factors affecting microvascular/nerve invasion in locally advanced gastric cancer. Microvascular invasion is one of the early metastasis modes of tumors.^[10]^ According to relevant reports,^[13]^ about 85% of N1-2 gastric cancer patients have a positive lymphovascular invasion, and about 30% of patients without lymph node metastasis have a negative lymphovascular invasion. In T1-2 stage gastric cancer patients, the positive rate of postoperative evaluation of lymphovascular invasion is about 28%, and it increases to about 80% in the T3-4 stage. In patients with stage N0 gastric cancer, the positive rate of perineural invasion is about 60%, and the incidence rate of lymph node-positive patients is 78%.^[14]^ Therefore, microvascular/nerve invasion may be a precursor to local tumor metastasis, and preoperative prediction of microvascular/nerve invasion may help identify high-risk patients and optimize preoperative treatment decisions. There is a correlation between microvascular/nerve invasion and the TNM stage of locally advanced gastric cancer, which is consistent with the results obtained in this study. TNM stage was selected through multivariate analysis of variance and found to be an independent predictor of microvascular/nerve invasion. CT is a noninvasive imaging examination method, with an accuracy of 80% to 89% and 63% for evaluating the T and N stages of gastric cancer, respectively.^[15]^ Therefore, predicting microvascular/nerve invasion status based on CT diagnosis of locally advanced gastric cancer TNM stage is feasible.

There is a correlation between the microvascular/nerve invasion status of gastric cancer and the degree of tumor differentiation.^[12,16]^ Duraker et al^[16]^ showed that patients with positive perineural invasion had significantly higher levels of low differentiation and lymph node metastasis compared to patients with negative perineural invasion. Liu et al^[12]^ believe that tumor differentiation is related to the occurrence of perineural invasion in advanced gastric cancer. This study also found that differentiation is an independent risk factor for microvascular/nerve invasion in locally advanced gastric cancer. Therefore, high attention should be paid to the degree of tumor differentiation in clinical practice, especially for locally advanced gastric cancer patients with a low degree of differentiation. Lauren classification is an independent risk factor affecting the prognosis of gastric cancer patients, with mixed type having the worst prognosis.^[17,18]^ There is currently limited research on the relationship between Lauren classification and microvascular/nerve invasion in gastric cancer, and some results are contradictory. A scholar^[12]^ analyzed the clinical data of 550 patients with advanced gastric cancer and pointed out that Lauren classification is an important indicator for evaluating perineural invasion in gastric cancer. The worse the Lauren classification, the stronger the tumor’s invasiveness, and the higher the probability of perineural invasion. De Franco et al^[19]^ also confirmed a correlation between perineural invasion in gastric cancer and Lauren classification. In addition, some scholars^[20]^ used energy spectrum CT multi-parameter imaging to evaluate the value of lymphovascular and perineural invasion in gastric cancer, and the results showed that there was no statistically significant difference in the proportion of Lauren classification between patients with positive and negative lymphovascular and perineural invasion.

Among the 325 locally advanced gastric cancer patients in this study, there were 128 male patients (67.72%) in the microvascular/nerve invasion group, significantly more than 61 female patients (32.28%). Multivariate analysis of variance showed that gender is not an independent risk factor for microvascular/nerve invasion in locally advanced gastric cancer, which is consistent with previous studies.^[21]^ In addition, studies have shown that body mass index, immunoglobulin A levels, and CT-based T and N stages can be used for predicting perineural invasion.^[22]^ In addition, studies^[22]^ have shown that low BMI (<18.5 kg/m^2^) is an independent prognostic factor for perineural invasion in gastric cancer, and further research is needed to explore the relationship between BMI and perineural invasion in more detail.

The results of the ROC showed that AUC values of TNM stage and differentiation were > 0.7, and the diagnostic efficacy of the combined parameter was better than that of the single parameter. AUC, sensitivity, and specificity were 0.819 (95%CI: 0.770–0.867), 66.7%, and 83.8%, respectively. High specificity is beneficial for early screening of microvascular/nerve invasion patients, therefore, combined parameter prediction of microvascular/nerve invasion has important value.

Our study had some limitations: (a) a single-center retrospective study, waiting for a multi-center prospective cohort study; (b) the sample size is insufficient to explore the influencing factors of VNI in locally advanced gastric cancer by increasing the sample size; (c) although standard CT scanning has been carried out and appropriate region of interest has been selected for measurement, it cannot completely correspond to pathological slices, and it needs to be determined jointly by pathology and surgeon.

## 5. Conclusions

In summary, TNM stage and differentiation are independent risk factors for microvascular/nerve invasion in locally advanced gastric cancer. If there are high-risk factors for microvascular/nerve invasion in locally advanced gastric cancer, it is recommended to develop a more aggressive treatment plan during the perioperative period.

## Author contributions

**Investigation:** Xiu-Chun Ren, Pan Liang.

**Methodology:** Pan Liang.

**Supervision:** Xiu-Chun Ren, Pan Liang.

**Writing – original draft:** Xiu-Chun Ren, Pan Liang.

**Writing – review & editing:** Xiu-Chun Ren, Pan Liang.
